# Sustained efficacy of closed loop electrical stimulation for long-term treatment of absence epilepsy in rats

**DOI:** 10.1038/s41598-017-06684-0

**Published:** 2017-07-24

**Authors:** Gábor Kozák, Antal Berényi

**Affiliations:** 1 MTA-SZTE ‘Momentum’ Oscillatory Neuronal Networks Research Group, Department of Physiology, University of Szeged, Szeged, H-6720 Hungary; 2 New York University Neuroscience Institute, New York University, New York City, 10016 NY USA

## Abstract

Closed-loop brain stimulation is a promising alternative to treat drug-resistant epilepsies. In contrast to optogenetic interventions, transcranial electrical stimulation (TES) does not require cellular modification of neurons to be effective, and it is less invasive compared to deep brain stimulation. Furthermore, on-demand TES of targeted brain regions allows the potential for normal function of these networks during interictal periods, a possibility that is eliminated by resective surgical treatment approaches. To further explore the translation of closed-loop TES for treatment of epilepsy, we show here for the first time that unsupervised closed-loop TES in rats can consistently interrupt seizures for 6 weeks and has the potential to control seizure activity up to 4 months (longest periods examined). On-demand TES significantly reduced the time spent in seizure and the individual seizure duration, although significantly higher seizure rate was observed during the treatment. The 6 week long stimulation had no residual adverse effects on the electrophysiologic characteristics of the brain after the termination of the treatment and did not induce glial remodelling in the brain. Our findings demonstrate the safety and effectiveness of minimally invasive, potentially lifelong TES treatment of epilepsy either alone or as a complement to drug treatments.

## Introduction

Epilepsy is a brain disorder that affects 1% of the population worldwide. Regardless of the intensive efforts to develop new pharmacotherapies^1^, antiepileptic drugs fail to adequately treat approximately one-third of the patients with epilepsy^2^, and even responsive subjects often suffer from side effects^3^. Surgical removal of epileptic foci is an option for some patients with medically-resistant epilepsy, but carries the risk of irreversible functional impairment. Thus, new therapeutic approaches are needed to overcome these shortages and a promising alternative is the on-demand epileptic seizure interruption^4, 5^. Various studies have developed methods to implement different intervention strategies in the recent years: i) optical^6–9^, ii) intracranial electrical stimulation^10^, and iii) transcranial electrical stimulation (TES)^11^.

Since optically driven seizure disruption requires the expression of genetically engineered foreign proteins by the targeted cells, there are substantial barriers to its use in the short term for human medical applications. Electrical stimulation techniques are more accessible, but require extensive investigation of the effects of time-targeted electrical perturbation of epileptic seizures in animal experiments. Importantly, most studies have not expanded beyond the acute effects of the treatment. Given the chronic nature of the epileptic process in the majority of patients, understanding of the long-term effects of a stimulation paradigm is critical. The few studies focusing on longer time scales involve exclusively intracranial electrical stimulation^10, 12–14^. This method has the disadvantage of being more invasive compared to the TES and more importantly, the specific targeting of intracranial electrodes requires the clear initial identification of a small number of key seizure choke points^15–20^.

TES has already been proven to effectively reduce the duration of spike-and-wave discharges in rats with thalamocortical epilepsy^11^, and a similar pattern is characteristic for absence seizures in human patients^21–23^. However, the clinical applicability of TES has not yet been realized, as this treatment effect was reported only on short timescales so far and it has been suggested that electrical treatments might be ineffective over longer timescales due to habituation^15, 16, 19^. Therefore, the main goals of the present study were to design a closed-loop intervention system, which continuously supervises brain activity for months and provides on demand seizure interruption for the early termination of seizures, and to determine whether the effective on-demand TES treatment of epileptic seizures over an extended period of time leads to a long-term therapeutic effect.

## Results

### Real-time detection of epileptic seizures

**Figure 1:**
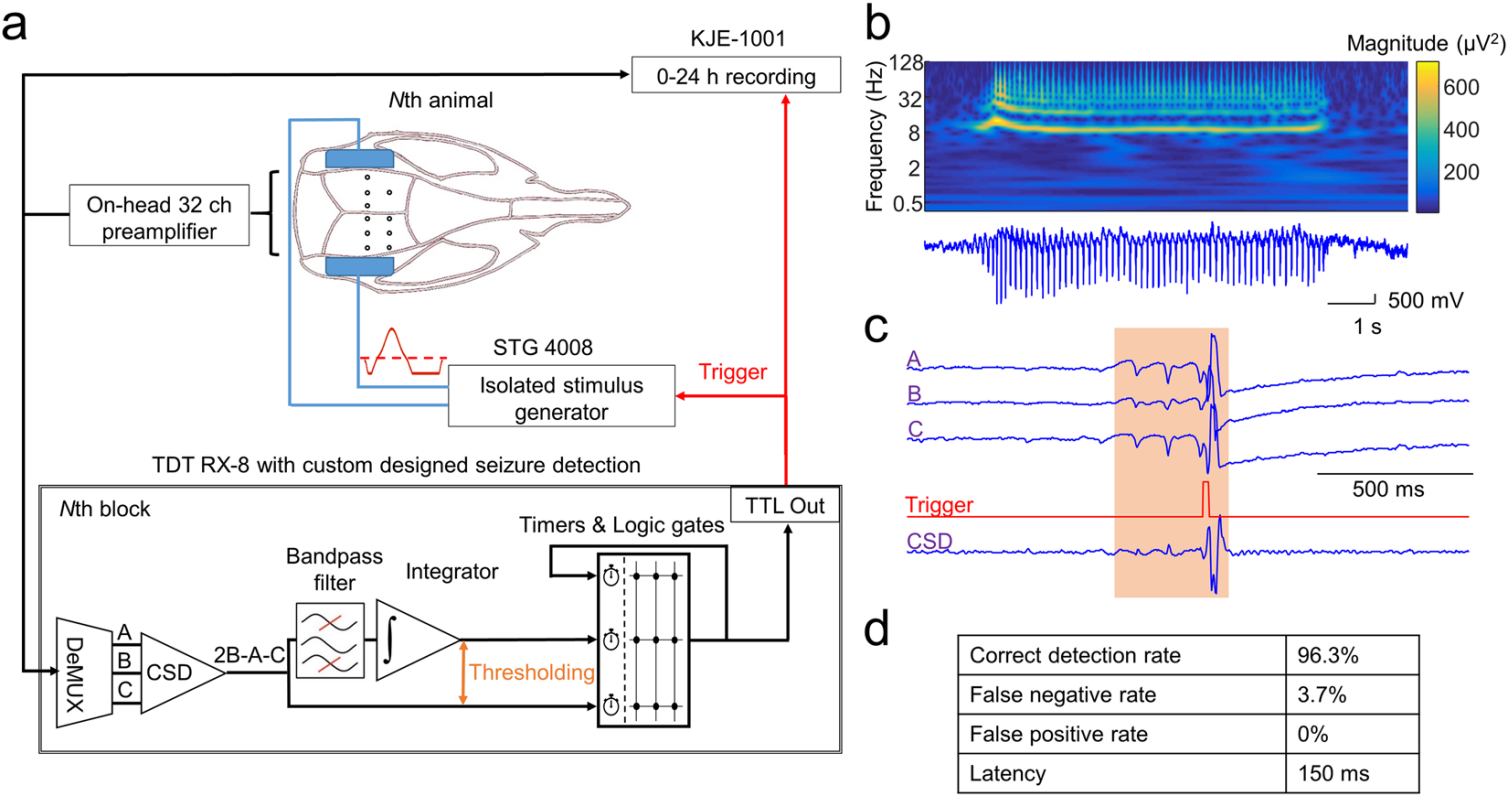
Overview of the closed loop system. (**a**) Cortical LFP from every rat (up to 8 simultaneously, max. 32 channel each) is amplified and multiplexed on-head, and fed to the digital signal processor (TDT RX-8) running a custom designed seizure detection algorithm. The signals were first digitally demultiplexed and CSD of one preselected tripolar electrode was calculated in each rat. Pre-set threshold crossings of the raw CSD and the bandpass filtered, integrated CSD was detected and in case of fulfilling the predetermined seizure criteria (coincidence, regularity) a single isolated, triphasic, charge neutral stimulus pulse was delivered. All the LFP signals and the on-line seizure triggers were continuously recorded for every rat. (**b**) Representative LFP trace and its corresponding wavelet spectrum of a spontaneous SW-discharges. (**c**) Representative LFP traces of a preselected tripolar electrode and the corresponding CSD during a spontaneously emerging interrupted spike-and-wave seizure. (**d**) Seizure detection performance of the closed loop system. Note the very low false negative and virtually absent false positive detections. Latency is determined solely by the seizure detection criteria, as the A/D conversion and the real-time DSP algorithm introduced only a few ms response delay.

We developed an unsupervised closed-loop intervention system to observe brain activity, and automatically terminate the epileptic seizures for durations comparable to the life expectancy of the animals (Fig. 1a, Methods). Briefly, intracortical recording electrodes and transcranial stimulating electrodes were implanted in Long-Evans rats (n = 13). All animals were showing the electrophysiological and behavioural symptoms of absence epilepsy, in the form of spontaneously emerging, rapidly generalizing, synchronous spike-and-wave (SW) episodes (Fig. 1b), accompanied with immobility, occasional whisker and head twitching and decreased sensory responsiveness^24–26^.

Seizures were detected based on specific ECoG parameters (Methods) and after each single detected SW-event a charge-balanced, triphasic stimulus pulse was applied transcranially to interrupt the ictal activity (Fig. 1c). To validate the performance of our seizure detection algorithm, in one animal we compared the seizure detection ability of the on-line algorithm to a manually refined semiautomatic detection in a 24 hours long recording session (Methods, Fig. 1d).

### Short-term closed loop seizure control

**Figure 2:**
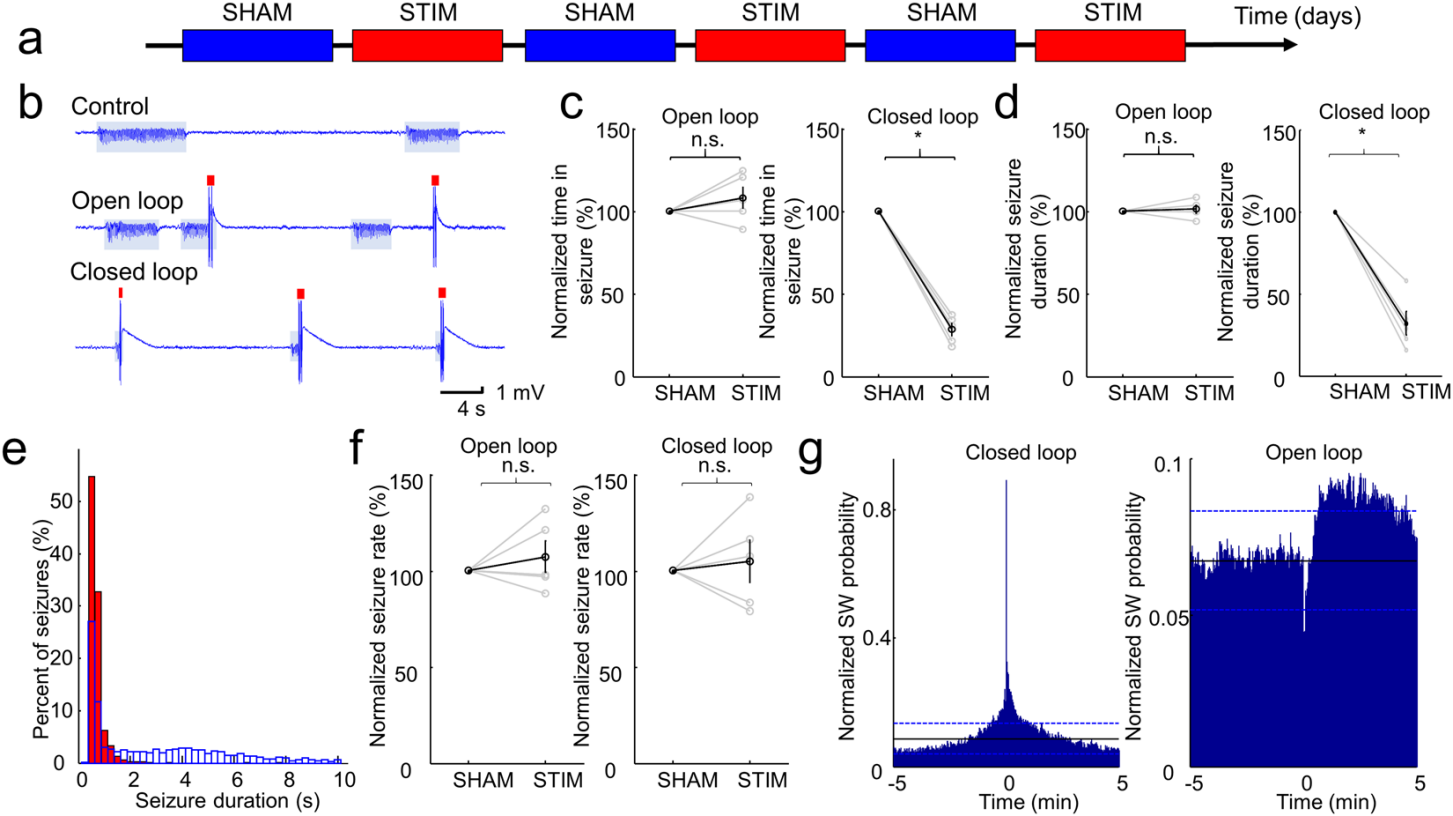
Results of the seizure suppression on the short term. (**a**) Experimental timeline showing a day-by-day alternation of sham and stimulated sessions. This pattern was repeated for 6 days. (**b**) Representative examples of LFP traces for the sham, pseudorandom and closed-loop seizure interruption protocols. Shaded bars and red ticks indicate the identified seizures and stimuli, respectively. **(c)** Time in seizure (normalized to the average seizure duration during the SHAM days for each animal) during SHAM and STIM days. Results from individual rats (grey lines) and group statistics (black lines) are shown. Stimulated day values are normalized to the preceding SHAM days’ values. Error bars represent ± SEM. (**d**) Average seizure duration (normalized to the average seizure duration during the SHAM days for each animal). Grey lines denote individual animals, while black lines represent the population data. Error bars represent SEM. (**e**) Representative example of the distribution of seizure durations during SHAM (blue) and STIM (red) days in one animal with closed-loop stimulation. (**f**) Seizure rates during the SHAM and STIM days (normalized to the average seizure rate during the SHAM days for each animal). Grey lines denote individual animals, while black lines represent the population data. Error bars represent SEM. (**g**) Peri-stimulus time histogram of the SW-complexes of a closed-loop and an open-loop stimulated rat across all the stimulated days. Black lines denote the average SW occurrence rate along the whole recording period and dashed blue lines refer to the confidence intervals (95%). Reference TES stimulus events are at time 0. *p < *0.01*, paired two-sample t-test (**c**), Wilcoxon rank sum test (**d**), paired two-sample t-test (**f**).

First, for validation purposes we demonstrated the short-term (day-long) effects of our seizure intervention system. Animals were divided randomly into three groups: control (n = 3), open-loop stimulated (n = 5) and closed-loop stimulated (n = 5) rats. The control animals were not stimulated at all, and the open-loop and closed-loop stimulated animals received treatment in an alternating fashion: each non-stimulated day (SHAM) was followed by a stimulated day (STIM) (Fig. 2a,b). The stimulation timing of every open-loop treated animal was driven by the stimulation timing of a randomly chosen closed-loop treated animal (known as yoked stimulation)^14^.

The overall time spent in seizure per day (Time in Seizure - TiS) only changed significantly in the closed loop stimulated group (5 of 5 animals showed a significant decrease, *p* < 0.05, paired two sample t test, STIM TiS was 33.0 ± 13.2% of SHAM TiS on the group level, *p* < 0.01, paired two sample *t*-test), confirming the effectiveness of the on-demand treatment on the short timescale (Fig. 2c). Furthermore, the stimulation significantly shortened the duration of the seizures in the closed-loop group (5 of 5 animals showed a significant decrease, *p* < 0.05, Wilcoxon rank sum test, STIM seizure durations were 32.1 ± 16.1% of SHAM seizure durations, *p* < 0.01, Wilcoxon rank sum test), but had no effect in the other group (Fig. 2d,e). These observations suggest that the timing of the TES is a critical factor in achieving an effective seizure control.

Stimulation, either open- or closed-loop protocols, had a variable effect on the occurrence rate of seizures (‘Seizure Rate’), but resulted no significant change on the group level (significant decrease in one of the open-loop treated animals (n = 5 rats) and significant increase in two of the open-loop and two of the closed-loop treated animals (n = 5 rats), *p* < 0.05, paired two sample t-test, no change on the group level, p = 0.2494 for open loop and p = 0.5418 for closed loop animals, Fig. 2f), pointing out the likeliness of a rebound effect after stimulation.

There was a marked, but not significant increase in the TiS for some open loop animals, but the mean seizure duration did not change in this group (Fig. 2c,d). These results raise the possibility that the randomly delivered stimulation may induce seizures, but it does not prolong them. Furthermore, considering the higher seizure rate that can be observed in some animals with the open-loop and closed-loop stimulation (Fig. 2f), we tested if the TES exerts a pro-convulsive effect. The peri-stimulus time-histogram of the SWs in the open loop animals revealed that following the random transcranial stimuli, the probability of SW occurrence is substantially lower than the baseline for approximately 30 seconds, but later the probability raises above the mean occurrence probability and can be even become significantly higher. In contrast to this, in case of the closed loop stimulation, the SW occurrence rate remains significantly higher following the first stimulus approximately for a minute, reflecting that the first stimulus cannot always stop the seizure activity or seizures may quickly recur in cases of incomplete termination. These findings suggest that there is no large direct seizure inducing effect of the stimulation and emphasize the crucial importance of the timing of the stimulation.

### Long-term closed loop seizure control

Next, we investigated whether long-term application of the closed-loop TES modifies the occurrence of the spontaneous seizures. Open-loop animals were merged into the control group, and one ‘closed-loop’ rat was substituted by a control animal due to his broken stimulating electrode. As a result, four control and five closed-loop stimulated rats were monitored for at least an additional 8 weeks continuously. The control rats did not receive any treatment during the observations. In case of the closed-loop stimulated rats the first week of the observation served as a control period (Pre-Treatment), then for 6 weeks the animals were stimulated in a closed-loop fashion (Treatment). After finishing the treatment, the rats were observed at least for one more week (Post-Treatment). Additionally, in one animal we continued the closed-loop treatment after the 8 week long experimental session for three more months with a week-long control session at the end.

**Figure 3:**
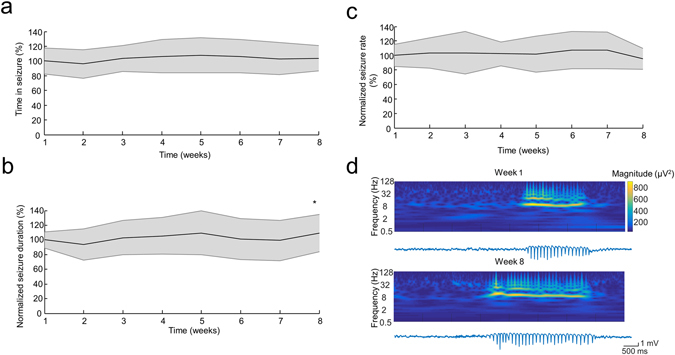
Seizure characteristics and signal quality of the non-treated animals. **(a,b** and **c)** Group data shows the Time in Seizure (**a**), average seizure duration (**b**) and seizure rate (**c)** over the weeks of observation (each measure is normalized to its corresponding mean ‘Week 1’ value for each animal). Shaded area represents ± SD. (**d)** Example LFP traces and continuous wavelet spectra of the recordings of one representative rat demonstrating the stable signal quality over the weeks. *p < *0.01 vs Week 2*, one-way ANOVA (**a,c**), Kruskal-Wallis test (**b**), both followed by *post hoc* HSD.

The control group (n = 4) did not show any significant change in terms of the TiS (one-way ANOVA, *F*_*(7,209)*_ = 0.95, *p* = 0.4665, Fig. 3a), in accordance with previous findings^24^. The normalized seizure duration (Kruskal-Wallis test, *χ* ^*2*^ = *15.9456*, *p* = 0.0256; Fig. 3b) was only significantly different between Week 2 and Week 8 (*post hoc* HSD, *p* = *0.0080*). The seizure rate did not change significantly during the weeks of observation (one-way ANOVA, *F*_*(7,208)*_ = 0.82, *p* = 0.5712, Fig. 3c). As the spectral composition of the SW waveforms (Fig. 3d) and the maximum amplitude of the SW patterns did not change, we concluded that the quality of the recordings did not deteriorate over the time course of the observations.

**Figure 4:**
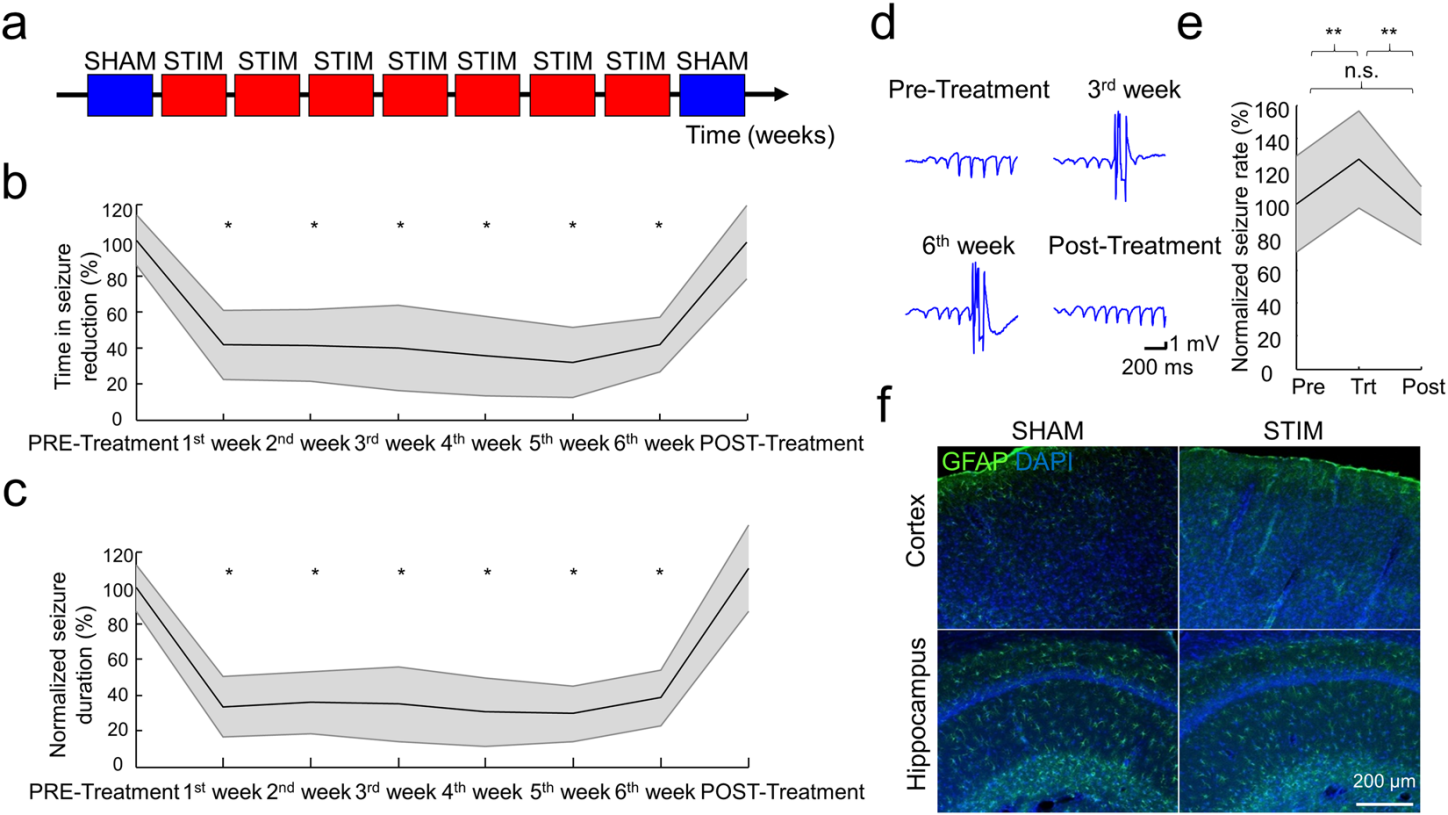
Results of the seizure suppression during the long-term stimulation protocol. **(a)** Experimental timeline. (**b** and **c)** Group data show the stable and similar decrease of Time in Seizure (**b**) and average seizure duration (**c**) as long as the closed-loop seizure suppression is on (each measure is normalized to its corresponding mean Pre-Treatment value for each animal). (**d)** Example LFP traces of one representative rat demonstrating the recording quality over the weeks. Note that the amplitudes and signal to noise ratios are qualitatively similar, suggesting a negligible change of the electrode conductance. (**e)** Group data show seizure rates before (Pre), during (Trt) and after (Post) the treatment (normalized to the average seizure rates during the Pre days for each animal). Shaded area represents ± SEM. (**f)** Representative histological examples of cortical and hippocampal regions of control (SHAM) and long term treated (STIM) animals, stained for GFAP (green) and DAPI (blue). Note that stimulation did not induce overt gross histological changes (i.e. gliosis) despite the long-term application. *p < *0.001*, **p < *0.05 vs Pre-Treatment*, one-way ANOVA (**b,e**), Kruskal-Wallis test (**c**), both followed by *post hoc* HSD.

We found that during the treatment all the animals (n = 5) spent significantly less time in seizure (38.8 ± 20.2%, mean across treatment weeks, one-way ANOVA, *F*_*(7,264)*_ = 72.47, *p* < 0.001, Fig. 3b), and the duration of the seizures significantly decreased (33.8 ± 17.8%, mean across treatment weeks, Kruskal-Wallis test, group *χ* ^*2*^ = *97.86*, *p* < 0.001, Fig. 3c). These effects were immediate and did not deteriorate over the time course of treatment (TiS = 41.8 ± 19.3% vs 41.9 ± 15.3%, *p* = 1.000; Normalized seizure duration = 33.4 ± 16.8% vs 38.6 ± 15.8%, *p* = 0.9932, for Week 1 vs Week 6, respectively, *post-hoc* Tukey HSD), and returned back to the Pre-Treatment level as the treatment was suspended (Post-Treatment TiS = 99.1 ± 20.4% of the Pre-Treatment TiS, *p* = 1.000; normalized Post-Treatment seizure duration = 111.3 ± 24.3% of the Pre-Treatment seizure duration, *p* = 0.5814, *post-hoc* Tukey HSD). The seizure rate was significantly increased in all animals during the treatment (133 ± 16.8% of the Pre-Treatment seizure rate, one-way ANOVA, *F*_*(2,9*)_ = 12.07, *p* < 0.05, Fig. 3e) and returned to the original level during the Post-Treatment period in 4 of 5 animals. One animal maintained a significantly lower seizure rate even after the treatment (75.86 ± 32.93% of the Pre-Treatment, *p* < 0.05, *post-hoc* Tukey HSD). Importantly, we did not observe any overt, major behavioural changes (alteration in nest building, rearing, sleep-awake cycle) or traces of glial remodelling due to the treatment (Fig. 4f), which is in accordance with the similarity of Pre- and Post-Treatment seizure parameters.

**Figure 5:**
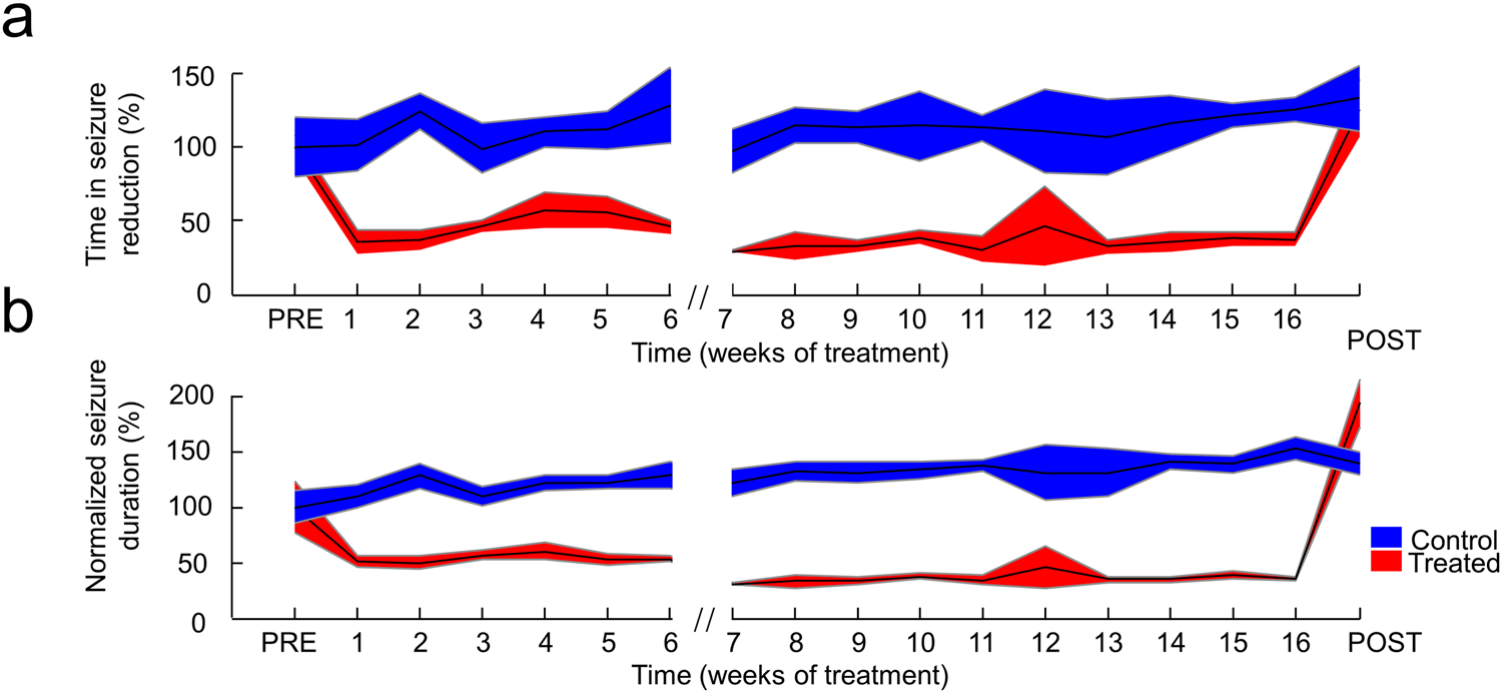
Sustained efficacy of the seizure suppression during the four-month long stimulation. **(a** and **b)** Results of an extended 4 months long treatment are similar to those obtained during the 6 weeks protocol (Fig. 4b and c), both for the Time in Seizure (**a**) and average seizure duration (**b**). Shaded area represents ± SD. (Control period at the end of the 6 week treatment is removed).

The prolonged closed-loop treatment (4 months) also resulted in qualitatively similar results, suggesting that lifelong TES treatment of epilepsy is possible, since the efficacy of the seizure suppression was maintained (Fig. 5a,b). After the termination of this four month long treatment, we observed that both TiS (133.1% ± 22.2% and 137.3% ± 21.2% of the Pre-Treatment TiS, for the control and the treated animal, respectively) and normalized seizure duration were substantially increased (138.8% ± 10.7% and 209.6% ± 23.5% of the Pre-Treatment seizure duration, for the control and the treated animal, respectively) in accordance with the trends observed in the control animal. This observation further supports that long-term closed loop treatment is possible in terms of the efficacy of the early termination of the seizures, but it may have no beneficial effect on the natural progression of the disease.

## Discussion

Our results provide evidence that TES can effectively control absence epileptic seizures in a closed loop fashion from days to months, and the treatment effect is instantaneous and does not deteriorate over time. Many attempts to prove the potential long term efficacy of electrical treatment of epilepsy have failed, possibly due to the lack of temporal selectivity^15, 19^ or due to selective intracranial targeting that misses the choke point locations of the epileptic network^15, 20^. The diffuse effect of the TES^27, 28^ may be remarkably beneficial in generalized seizures, where the pattern generation is the result of a distributed network even if the initiation is triggered from a specific location^29, 30^.

In this study we investigated spontaneously emerging absence seizures^31, 32^, which is a widely used epilepsy model to test different non-pharmaceutical treatment approaches in rodents^33^. Only male animals were included in our experiments and to date there is no available information about sex differences regarding seizure properties of the Long Evans strain^34^. Although this is a considerably mild form of epilepsy, owing to the high spontaneous seizure rate it is an ideal model to investigate the seizure – stimulus interactions^35^. It is not possible to directly draw conclusions how other generalized seizure types would respond to TES treatment, nevertheless it is promising that in a particular type of generalized epilepsy it is feasible to control seizure activity on the long term. Clearly, further studies are necessary to test TES on other seizure models to reveal the full potential of this treatment, such as temporal lobe epilepsy, which is the most common type of seizures in the adulthood^36^.

Previous papers describing an increased seizure frequency during electrical suppression of absence seizures suggest^16, 37^ that this phenomenon may be related to the very high spontaneous seizure rate of this epilepsy model. Nonetheless, this side effect of the closed loop stimulation has to be taken into account when considering the feasibility of the clinical application, as the possible seizure rate increase in response to the treatment may be a potential limiting factor determining the tolerance of the patients.

Spike-and-wave discharges are most likely to occur during periods of slow-wave sleep and drowsiness^32^. Previous studies reported that antiepileptic stimuli do not change the behavioural state of the animal^16^ and do not induce arousal^11^, therefore the animal – when the seizures are stopped – remains in the seizure susceptible behavioural state. Consequently, the higher seizure rate may be coming from the fact that the due to the early termination, there is more room for further seizures within the given period when the brain is more prone to develop seizures.

Furthermore it cannot be excluded, that the recurrence of suppressed seizures reflected by the increased seizure rate may be the result of incomplete switching between the firing modes of the thalamocortical cells^18^, leading to a rebound phenomenon^15, 19^. Importantly, these rebound or recurrent seizures did not increase the total time spent in seizure, as the shortening of the average seizure duration more than compensated the increase in the seizure rate, thus altogether it decreased significantly (Figs 2d and 4b).

It is long known that the cells of the thalamus can express various response patterns depending on their firing modes^23^. Consequently the strikingly different effect of the random and closed-loop TES can be explained by this state-dependent response of the thalamocortical loop. Stimulation during the already hypersynchronous state (closed-loop stimulation) may force neurons to desynchronize if an early stimulus induced excitation prevents them generating low-threshold Ca^2+^-spikes during the duty cycle of SW-discharge^11, 38^. On the contrary, in a random initial state, the external stimulus may weakly synchronize cells making them susceptible to randomly reach a level of synchrony and initiating a seizure, which leads to an indirect increase of seizure rate. This state-dependency of the outcome underlies the importance of timing.

An important conclusion of our experiments is that the seizure-related hypersynchronous electrical activity is not required for the maintenance of seizure potential, as we could not achieve a curative effect even if the seizures were suppressed for months, although our treatment did not prevent, only shortened the seizures. Similarly to the deep brain stimulation (DBS) for Parkinson’s disease (PD)^39, 40^, the investigated seizure parameters returned to the baseline as we stopped the treatment in most of the animals, suggesting that the effect does not cumulate, and the seizure generating networks do not habituate, which parallels the lack of histological alteration (Fig. 4f). Furthermore, the closed loop TES was not able to stop the progression of the disease in the prolonged closed-loop treatment either, despite the effective seizure control during this period (Fig. 5a,b). Note that similar observations were reported regarding the lack of lasting DBS effect in PD^41^, as it does not stop the death of dopaminergic neurons, but rather treats the symptoms of the disease^41, 42^. In contrast to this, we cannot yet exclude that the reason for the lack of the curative effect in our epilepsy related TES application is due to the fact, that the closed loop detection requires a seizure to be generated, thus the seizures were not prevented but only reversed. There are efforts to predict the seizure emergence^33, 43^ and if reliable predictors can be identified, it may ultimately clarify the therapeutic effects of TES when used for preventing and not only terminating the seizures.

Very often the general condition of patients with epilepsy deteriorates over time due to the overexcitation of the microcircuitry and cell death, leading to memory impairment, decline in intellectual performance and a higher risk of sudden unexpected death in epilepsy (SUDEP)^44–46^. The main effect of TES according to this dataset — namely the sustained lower time spent in seizure and shorter seizures during treatment — might have the potential to attenuate or prevent the secondary consequences of epilepsy, but it requires further investigations. Our results suggest, that TES might have a favourable impact on the long term outcome of the disease, even if it cannot achieve a cumulative effect regarding the seizure activity.

Importantly, effective seizure control of epileptic patients can improve their quality of life^47^, even if the treatment is not able to cure the underlying mechanism of epilepsy. There are available comparisons about the happiness, self-esteem and other psychosocial parameters with and without successful treatment^48^, showing a clear correlation between the effectivity of treatment and general well-being of the patients, but the importance of terminating seizures as soon as possible and reducing the non-responsive periods to avoid accidents (e.g. driving vehicles, handling machinery, etc.) is probably even larger. Therefore, many secondary positive effect might be expected from closed-loop TES treatment besides those which can be tested in animal models.

These findings demonstrate the safety and effectiveness of TES treatment, and suggest that it could be used as a minimally invasive, lifelong palliative treatment of certain types of epilepsy, alone or as a complement to pharmaceuticals. To date, short-delay, system-on-a-chip solutions are already available for real-time epileptic seizure detection tested in animal models^33^, which can be combined with reliable detection algorithms optimized for human data^49, 50^. In addition, brain-machine-brain interfacing is feasible through medically approved non-penetrating electrodes. Therefore every aspects are given to translate our previous and current results on the proof of the concept and safety of a temporally targeted on-demand transcranial seizure suppression approach into a potentially effective, nonpharmaceutical antiepileptic therapy for human patients.

## Methods

### Animals

All experiments were performed in accordance with European Union guidelines (2003/65/CE) and the National Institutes of Health Guidelines for the Care and Use of Animals for Experimental Procedures. The experimental protocols were approved by the Ethical Committee for Animal Research at the Albert Szent-Györgyi Medical and Pharmaceutical Center of the University of Szeged (XIV/218/2016). Thirteen male Long-Evans rats (300–640 g) were used in the present study.

### Surgery

The animals were operated under isoflurane anaesthesia and chronically implanted with intracortical recording and transcranial stimulating electrodes according to the following procedure: Tripolar electrodes were prepared to record neocortical and hippocampal local field potentials (LFP). Three 50-μm diameter polyimide-insulated tungsten wires (Tungsten 99.95%, California Fine Wire, Grover Beach, CA, USA) were inserted into a 180-μm inner diameter stainless steel tube (Vita Needle Company, Needham, MA, USA), and their tips were spaced 400 μm vertically from each other. Impedances of the wire electrodes varied between 30–90 kΩ at 1 kHz.

Rats were implanted with ten (n = 6), seven (n = 5) or six (n = 2) tripolar electrodes (30, 21 and 18 recording channels, respectively), which targeted the frontal and parietal cortical areas of both hemispheres and over the right hippocampus. The electrodes were vertically advanced through individual holes in the skull, until the most superficial wire of the electrode reached the surface of the cortex. The holes around the electrodes were filled in with non-conductive silicone (Dow Corning Corporation, Auburn, MI, USA) and the electrodes were fixed carefully with dental cement (Duracryl™ Plus, Spofa Dental, Jičín, Czech Republic). Miniature stainless steel screws (serving as reference and ground) were implanted above the cerebellum.

Transcranial stimulating electrodes were prepared as follows: two strips of three plastic pockets containing densely coiled stripped wires (Phoenix Wire Inc., South Hero, VT, USA) were glued onto the temporal bone bilaterally, using cyano-acrylic glue (Loctite, Henkel, Germany). Pockets were filled with conductive paste (Super Visc, Brain Products, Gilching, Germany) and were electrically isolated with cyanoacrylic glue and dental cement from the tissues. Unilateral pockets were wired together serving as a single pole.

### Electrophysiological recordings and stimulation

All recording sessions took place in the same room in 12 h light-dark cycles. The rats were housed individually in plastic cages (42 × 38 cm, 18 cm tall), the walls were made of clear plexiglas and food and water were given *ad libitum*. After recovery from the surgery (minimum 3 days), the rats were connected to the closed-loop recording and intervention system and were observed continuously for at least 3 months continuously. All animals were initially exposed to the short-term (6 days) stimulation protocol, and then sequentially to the long-term stimulation protocol. To avoid the twisting and overtension of the cables, a very thin, lightweight recording cable (40 AWG Nylon Kerrigan-Lewis Litz wire, Alpha Wire, Elizabeth, NJ, USA) and a suspended commutator (Adafruit, New York, NY, USA) sliding vertically on guide rails with the help of a counterweighted trolley system were used.

The recorded signals were preamplified (n = 30, 21 or 18 channels/rat), amplified 400×, multiplexed on head and stored after digitalization at 500 Hz sampling rate per channel (KJE-1001, Amplipex, Szeged, Hungary). Simultaneously, the preamplified signals of all parallel recorded rats (up to 8) were analysed on-line by a programmable digital signal processor (RX-8, Tucker-Davis Technologies, Alachua, FL, USA) using a custom made seizure detection algorithm, as follows.

The LFP of pre-selected tripolar electrodes were demultiplexed (one triplet for each rat) and the current source density (CSD) of those triplets were calculated [(CSD = 2 × intermediate − (deep + superficial electrode))]^11^. The manual selection of the triplet was based on the consideration of the anatomical location of the electrodes, earliest seizure appearance and the signal-to-noise ratio of the triplets. The derived signal was bandpass-filtered, rectified and integrated in a time window of 20 ms (reflecting the monotonicity of the signal within the temporal window). Threshold crossing for both the raw CSD and the integrated signal (coincidence) were monitored. Synchronous multiple threshold crossing (minimum 2, separated by 40–50 ms, regularity) triggered a charge neutral, triphasic single-pulse (100 ms) stimulation (STG4008; Multi Channel Systems, Reutlingen, Germany). The thresholds of the detection algorithm were set for each rat separately and were periodically fine-tuned based on the variance of the signal, when it was necessary. The stimulation was performed either in voltage or current controlled mode, the stimulus intensity was titrated until a reliable seizure-stopping effect was observed (approx. 1.5–2 mA in all animals). The generated intracranial electrical gradient was approximated as the stimulus induced voltage difference measured between multiple recording triplets divided by the intertriplet distance. During the long-term observations, the stimulus intensity was regularly re-adjusted to maintain the same intracranial gradient.

### Histology

For histological verification of the recording locations and possible pathologic changes, i.e. stimulus induced gliosis, animals were deeply anaesthetized with 1.5 g/kg urethane (i.p.) and transcardially perfused with saline followed by 4% paraformaldehyde and 0.2% picric acid in 0.1 M phosphate buffer saline. After overnight postfixation, 50-μm thick coronal sections were prepared with vibratome. The sections were then either immunostained for GFAP with DAPI counter (Millipore Cat# MAB360, RRID:AB_2109815), or subjected to cresyl violet staining with standard histological techniques^51^. GFAP signalling were examined with a Zeiss laser scanning microscopy (LSM880) from sections every 1 mm throughout the entire anterior–posterior extent of the cerebrum.

### Off-line analysis

Off-line detection of the epileptic activity was performed in a similar way to the online method. SW episodes were detected from the off-line calculated CSD signals. The LFP was band-pass filtered with a 4th order zero phase lag Butterworth filter between 8 and 12 Hz (low BW) and 30 and 200 Hz (high BW), and the peaks of the spike components were detected if the high BW signal and the low BW signal conjunctively exceeded +5 standard deviation (SD) of their corresponding baseline activity. The results of the automated algorithm were manually revised and the threshold of the spike detection was manually re-adjusted in cases when the automatic detection generated false positive or false negative events. Consecutive SW episodes were considered separate if at least a 1-sec gap was present between them, otherwise they were merged. Events containing less than 3 spike components (shorter than 0.3s) were excluded from the analysis. Continuous wavelet spectra and power spectra were calculated in Matlab using Wavelet Toolbox and Chronux Toolbox (http://chronux.org/), respectively^52^.

### Statistical analysis

Statistical tests were performed in Matlab using the Statistics Toolbox. Results are expressed as mean ± standard deviation (SD), unless stated otherwise. Differences between time spent in seizure for SHAM and STIM periods in the short term experiments were compared using paired two sample t-test. Seizure rates were compared using a paired two sample t-test in the short term experiments and one-way repeated measure ANOVA in the long term experiments. Statistical significance of differences between time spent in seizure for Pre-Treatment, Treatment and Post-Treatment periods were evaluated using one-way repeated-measure ANOVA at both individual and group level in the long term experiments. Effects of the treatment on the seizure duration in the short term was tested using Wilcoxon signed rank sum test, as for the chronic condition using Kruskal-Wallis one-way analysis of variance. For multiple comparisons Tukey’s honesty post-hoc test was employed. Alpha was set to 0.05, all t-tests were two tailed. All datasets met the criterion of normal distribution where parametric tests were used.

Peri-stimulus time-histograms (PSTH) were constructed with the SW events [−5, 5] mins around TES stimuli with a bin size of 2 seconds. For each animal the histograms of the different days were summed up. 95% confidence intervals were determined from the pre-stimulus periods 5 minutes prior to the stimuli based on the confidence limits of Poisson distribution^53, 54^. Bins, of the original PSTH exceeding the boundaries of the confidence interval, were identified as significant bins.

### Data availability

The datasets generated and analysed during the current study are available from the corresponding author on reasonable request.
